# Relative contribution of photodegradation to litter breakdown in Australian grasslands

**DOI:** 10.1002/ece3.10710

**Published:** 2023-12-06

**Authors:** Freja E. B. Butler, Megan K. Good, John W. Morgan, Nick L. Schultz

**Affiliations:** ^1^ Future Regions Research Centre Federation University Australia Ballarat Victoria Australia; ^2^ Ecology Australia Thomastown Victoria Australia; ^3^ School of Agriculture, Food and Ecosystem Sciences The University of Melbourne Melbourne Victoria Australia; ^4^ Department of Environment and Genetics La Trobe University Bundoora Victoria Australia

**Keywords:** carbon cycling, litterbag, *Poa labillardierei*, recalcitrant litter, *Themeda triandra*

## Abstract

Grassy ecosystems cover ~40% of the global land surface and are an integral component of the global carbon (C) cycle. Grass litter decomposes via a combination of photodegradation (which returns C to the atmosphere rapidly) and biological decomposition (a slower C pathway). As such, decomposition and C storage in grasslands may vary with climate and exposure to solar radiation. We investigated rates of grass litter decomposition in Australian temperate grasslands along a climate gradient to uncouple the relative importance of photodegradation and climate on decomposition. Litterbags containing leaf litter from two common native grass species (*Poa labillardierei*, *Themeda triandra*) were deployed at six grassland sites across a precipitation gradient (380–890 mm) in south‐eastern Australia. Bags were retrieved over 39 weeks to measure mass loss from decomposition. We used shade treatments on the litter of one species (*T. triandra*) to partition photodegradation from biological decomposition. The shade treatment reduced the rate of decomposition of *T. triandra* relative to the full‐sun treatment at all sites, by an average of 38% at 39 weeks; the effect size of the shade treatment was not correlated with site productivity. The rate of decomposition in both species was positively correlated with rainfall midway through the experiment, but there were no significant differences in total decomposition among sites after 39 weeks. By week 39, total decomposition of *T. triandra* was significantly greater than for *P. labillardierei*. In general, we observed relatively linear decomposition rather than the strong negative exponential decay observed in many global litter decomposition studies. *Synthesis*: We found that solar radiation exposure was a strong contributor to litter decomposition in temperate Australian grasslands across a broad climate gradient, which may be related to a period of photopriming prior to further biotic decomposition. This study highlights the importance of litter composition and solar radiation exposure in our understanding of how decomposition patterns contribute to global C cycling.

## Graphical abstract

We investigated rates of grass litter decomposition in Australian temperate grasslands along a climate gradient to uncouple the relative importance of UV radiation and climate on decomposition. We found UV exposure to be a strong contributor to litter decomposition, regardless of climate. This may be due to the recalcitrant nature of the litter used, highlighting the importance of litter quality in determining decomposition patterns.

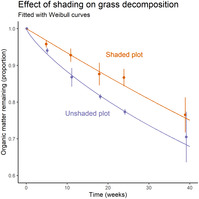

## 1 INTRODUCTION

In terrestrial ecosystems, plants sequester carbon (C) from the atmosphere via photosynthesis and C is then released through litter decomposition (Montaña et al., 1988; Olson, 1963). Abiotic litter decomposition processes such as photodegradation and thermal degradation can rapidly return C to the atmosphere as volatile C compounds (Brandt et al., 2009; Lee et al., 2012). By contrast, a larger proportion of C is stored as soil organic matter if microbes and other soil organisms are the primary mode of decomposition (Austin & Vivanco, 2006; Robertson & Paul, 2000; Rutledge et al., 2010). The relative contribution of different decomposition pathways in any ecosystem is influenced by interactions among abiotic and biotic processes (Austin & Vivanco, 2006; Brandt et al., 2010; Montaña et al., 1988) and hence, a mechanistic understanding of decomposition pathways in different biomes is essential for estimating global C cycle dynamics.

Grasslands cover approximately 40% of the Earth's land surface (Gibson, 2009) and make an important contribution to the global C balance, accounting for around 20% of the total C in soil and vegetation (Adams et al., 1990). Litter decomposition is well‐studied in grasslands of the northern hemisphere (e.g. Bontti et al., 2009; Brandt et al., 2010; Henry et al., 2008; Liu et al., 2010; Smith & Bradford, 2003; Vossbrinck et al., 1979; Zhang et al., 2008), but this understanding may not translate to grasslands globally. Many fundamental questions about litter decomposition in grassy ecosystems are unresolved, including how decomposition is influenced by water limitation (Austin & Vivanco, 2006; Baker & Allison, 2015), climate variability (Weatherly et al., 2003), litter quality (Brandt et al., 2007; Couteaux et al., 1995; Meentemeyer, 1978) and soil nutrient status (Ge et al., 2013).

Litter decomposition has not been well‐studied in Australian grasslands. Australia's rainfall is the most variable of any landmass (Nicholls et al., 1997; Risbey et al., 2009) and experiences higher summer ultraviolet (UV) levels than the equivalent latitudes in the Northern Hemisphere (Gies, 2003). Additionally, Australian grassland soils have the lowest nitrogen (N) and phosphorus (P) content of any global grasslands (Fay et al., 2015), and such factors can affect litter quality (Aerts, 1997).

Whilst growing conditions have been shown to influence litter quality (Aerts, 1997), Osanai et al. (2012) and Vivanco and Austin (2006) demonstrate that different species grown under the same conditions can have intrinsic differences in litter quality. Indeed, in a meta‐analysis by Cornwell et al. (2008), species‐specific differences in litter decomposability were found to be related to the species' ecological strategy. Photosynthetic pathways utilised by different species can also influence a variety of plant characteristics including litter quality (Vivanco & Austin, 2006). Given the substantially different growing conditions in Australia, coupled with a unique flora—with high levels of plant endemism present (Bryceson & Morgan, 2022; Crisp et al., 2002)—it is expected that Australian native grass species (and potentially non‐native species grown in Australia) will exhibit different litter quality and decomposition trends to grass species elsewhere globally.

Litter quality (i.e. its chemical and morphological characteristics) is a strong predictor of both the rate and pattern of decomposition (Cornwell et al., 2008; Couteaux et al., 1995; Meentemeyer, 1978; Zhang et al., 2008). High concentrations of nutrients in leaves (such as N and P) increases mass loss from labile molecules in the early stages of decomposition (Gill et al., 2022). Recalcitrant molecules, such as lignin, however, are slow to decompose due to their large and complex structure (Coleman et al., 2004; Ruiz‐Duenas & Martinez, 2009). Furthermore, whilst N promotes the initial decomposition of labile molecules, it can have an inhibitory effect on subsequent stages of litter decomposition (Couteaux et al., 1995; Gill et al., 2022). These factors lead to rapid early‐stage decomposition and slower late‐stage decomposition and hence, to the negative exponential decay curve exhibited in many decomposition studies (Couteaux et al., 1995; Olson, 1963). However, Cornwell and Weedon (2014) posit that the negative exponential model does not explain the empirical evidence presented in many studies, such as when there is a lag phase of slow early‐stage decomposition, or when N concentration is lower. As such, in Australian native grasslands where climate, soils and evolutionary history are different to northern hemisphere counterparts (Orians & Milewski, 2007) we might expect a more linear decomposition trajectory or a lag phase during early decomposition.

Climate influences decomposition, with rainfall and temperature acting at regional scales to provide the overarching conditions for long‐term decomposition rates (Berg et al., 1993; Meentemeyer, 1978). Photodegradation also plays an important yet somewhat unresolved role in litter decomposition (Austin & Vivanco, 2006; Brandt et al., 2010; Gallo et al., 2009; Gaxiola & Armesto, 2015; Henry et al., 2008). In contrast to microbial decomposition, photodegradation is not limited by water availability (Gallo et al., 2009). Photodegradation of lignin can also remove chemical and physical barriers to decomposition, increasing subsequent biotic decomposition (Austin et al., 2016). However, the relative role of photodegradation along a gradient of moisture availability is debated. Brandt et al. (2007) and Almagro et al. (2016) found photodegradation to be particularly significant for decomposing litter at sites with low moisture availability, although Brandt et al. (2010) found that the proportional contribution of photodegradation to decomposition was not greater at drier sites. Hence, the relative role of photodegradation requires testing across productivity gradients, for different grass species and plant traits, and in different environments.

The native grasslands of south‐eastern Australia occur over a broad climate gradient and are an ideal place to test some of the key drivers of litter decomposition. In this study, we compared the decomposition patterns of two perennial grass species (with different photosynthetic pathways) at grassland sites along a climatic gradient.

Therefore, based on what we know of litter decomposition elsewhere, we predicted that (1) decomposition will vary among grass species, (2) higher rainfall sites will have higher rates of litter decomposition and (3) we will not observe a strong negative exponential decomposition trajectory due to the low nutrient status of Australian grasses. Finally, by applying shade treatments to a subset of plots across all sites, we expected to find that photodegradation will contribute significantly to litter decomposition rates in Australian grasslands.

## 2 MATERIALS AND METHODS

**FIGURE 1:**
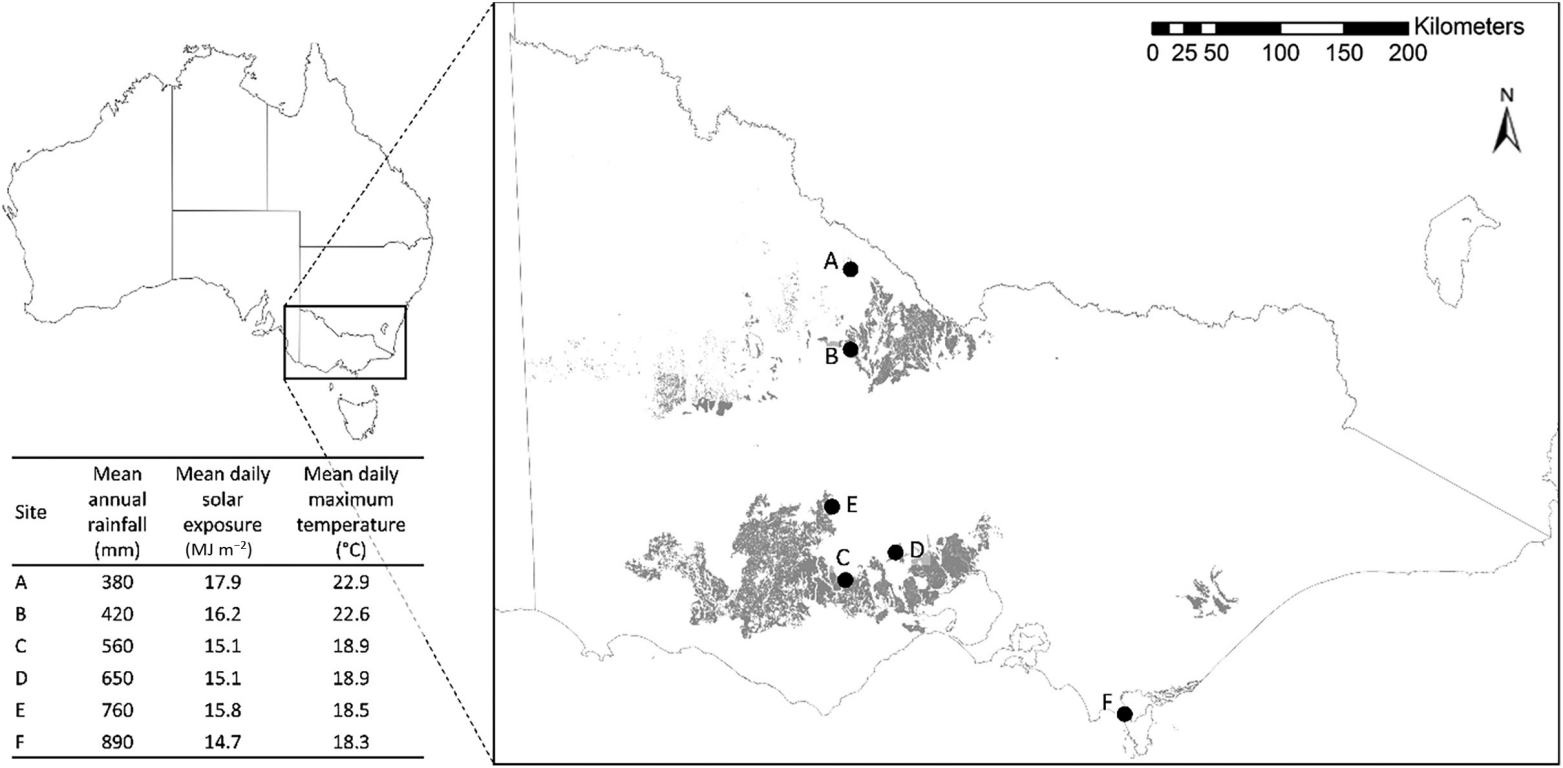
Location of six study sites across Victoria, Australia. Total rainfall (mm), mean daily solar radiation (MJ m^−2^) and mean maximum daily temperature (°C) for each site during the study period are shown (*Climate data source*: SILO Long Paddock, Queensland Government, 2020). The study period ran from 5/11/2018 to 12/8/2019. The grey‐shaded area is the natural distribution of Plains Grassland. *Data source*: Data Vic (2008) NV1750_EVCBCS.

This study aimed to test the role that climate, litter type and photodegradation play in litter decomposition of dominant native tussock grasses from the temperate grasslands of south‐eastern Australia (35–39° S, 143–147° E). We focused on six native grasslands distributed across gradients of mean annual rainfall, temperature and solar exposure (Figure 1). All sites experience winter‐dominant rainfall. Soils varied, with sodosols at sites A, B and C, kurosols at site D, dermosols at site E and rudosols at site F (Data Vic., 2020). The southern, higher rainfall sites (sites C, D, E, F) were dominated by the perennial tussock grasses *Themeda triandra* (C4), *Anthosachne scabra* (C3) and *Poa labillardieri* (C3) whereas the northern, lower rainfall sites (sites A, B) were dominated by the perennial tussock grasses *Austrostipa* spp. (C3), *Rytidosperma* spp. (C3) and *Enteropogon acicularis* (C4).

To test the effects of litter type on decomposition, one C3 grass (*P. labillardierei* var. *labillardierei*) and one C4 grass (*T. triandra*) was selected for study, and recently senesced leaf litter that was still attached to the tussock was collected from the field in October–November 2018 (austral spring). Litter was oven‐dried at 40°C for 48 h. Three grams of dry leaf litter was placed in 15 × 15 cm litterbags made from nylon mesh with a 1.5 mm aperture, and the tops of the bags were folded over and stapled. A total of 360 litter bags were produced: 240 containing *T. triandra* (the dominant grass of mesic grasslands; C4) and 120 containing *P. labillardierei* var. *labillardierei* (a dominant grass in wetter grasslands; C3). The total weight of the litterbag and its contents were recorded prior to deployment. Dry samples of each grass species were analysed for C and N content using the Dumas combustion method (Dumas, 1831), and lignin was quantified using the acid detergent lignin method (Van Soest, 1967).

### 2.1 Experimental design

**FIGURE A1:**
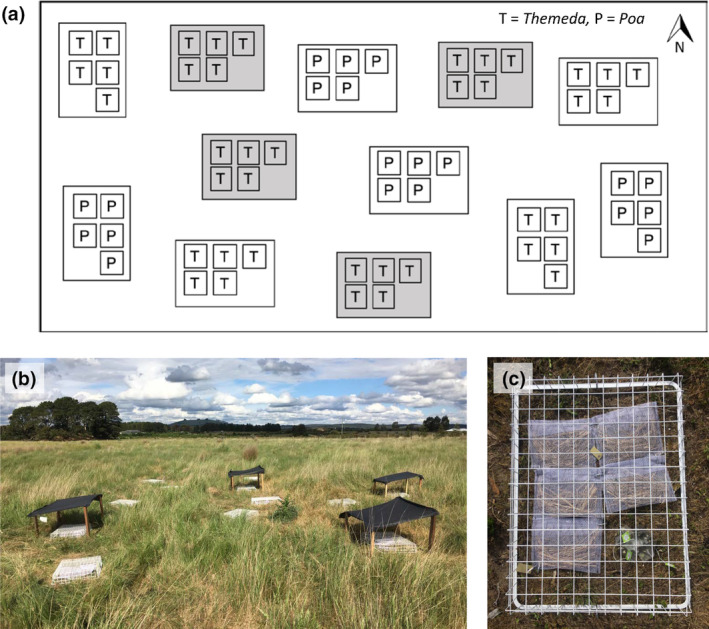
(a) Scheme of experimental design at each site. Plots shaded grey represent those with a shade structure. (b) Photograph of a site after setup, showing cages and shade structures. Shade structures were angled towards the North to increase shading of litterbags throughout summer. (c) Photograph of one of the cages with deployed litterbags. Teabags are also visible (bottom right‐hand corner; data not reported in this paper).

Litterbags were deployed in late austral spring of 2018, towards the end of peak growing season for C3 grasses (Groves, 1965). At each of six grasslands, a 5 × 5 m area was chosen and twelve 1 × 1 m plots (four *P. labillardierei* var. *labillardierei* plots in full sun and eight *T. triandra*, of which four were shaded and four were in full sun) were haphazardly located for deployment of litter bags; plots were a minimum of 1 m apart (see Figure A1). All plots were clipped to ground‐level to allow the litterbags to be placed directly on the ground and to minimise shading by neighbouring tussocks. In each of the 12 plots at each site, five litterbags were deployed. Each plot was protected from animal disturbance by a small rigid wire cage (9 cm high) held down by pegs. We erected solar radiation interception shelters using shade cloth attached to structures that extended 10 cm beyond the margins of the wire cages, over four of the eight *T. triandra* plots at each site. Shade cloth was angled towards the north (the source of prevailing incoming solar radiation in austral summer), which prevented rainwater pooling on the shade cloth and dripping onto litterbags. The shade cloth was 30–45 cm above the top of the wire cages to minimise increasing humidity around the litterbags. The shade structure was not designed to completely block solar radiation, and the litterbags underneath the shade structures were not shaded for the entire day. UV readings at midday in full sun showed that, across all sites, the shade treatment reduced UV radiation by 94.9% ± 0.6%. UV measurements taken at the same time under grass tussocks showed that their shade reduced UV radiation by 92.9% ± 0.8% across all sites, suggesting that the effect of the shade treatments on UV was comparable to the shade provided by perennial tussock grasses.

Litterbags were retrieved on five occasions after deployment (at weeks 5, 11, 18, 24 and 39), spanning austral summer through to winter. One litterbag was retrieved from each five‐bag plot on each sampling occasion. Litterbags were oven‐dried for 48 h at 40°C and weighed to measure mass loss from decomposition. Loose soil was encrusted onto some litterbags bags, and this was gently removed prior to weighing.

### 2.2 Data analyses

We used the proportion of organic matter remaining (OMR) to represent the amount of decomposition that had occurred in each bag at each sample period, calculated as:

$$\mathrm{OMR}=\frac{m_{i}-m_{f}}{m_{i}}$$

where *m*_*i*_ is the initial dry mass of litter in each bag, and *m*_*f*_ is the final dry mass of litter after.

**FIGURE A2:**
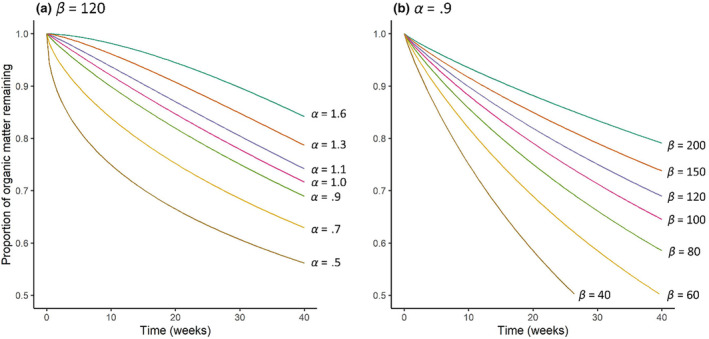
Examples of Weibull curves showing the effect of *α* and *β* constants on the shape of the curves. These curves are based on the timeframe (40) weeks and the magnitude of organic matter loss observed in this study.

We fit proportion of OMR for each site × species combination to three models for comparison: a Weibull residence model, a negative exponential model and a linear model. We fit the Weibull residence model sensu Cornwell and Weedon (2014):

$$F\left(t,\alpha ,\beta \right)=e^{-{\left(\frac{t}{\beta }\right)}^{\alpha }}$$

where α is a constant that controls the shape of the decomposition trajectory, β is a scaling parameter that reflects the rate of decomposition, and t is time. When α ~ 1, the decomposition rate is constant through time and the curve is akin to a standard exponential decay model. If α > 1 it indicates a lag in the beginning of decomposition, and when α ≪ 1, there is a high decomposition rate early in the decomposition process relative to later stages. Figure A2 shows the influence of α and β values on the shape of decomposition curves. Cornwell and Weedon (2014) found that the Weibull model suits a broader range of decomposition trajectories, including those with an initial lag phase, than the more‐commonly used negative exponential model and other alternative models.

The negative exponential model (Olson, 1963) is commonly used in decomposition studies. This model assumes a constant rate of decay:

$$\mathrm{OMR}=e^{-\mathrm{kt}}$$

where *k* is the decay constant and *t* is time in days. We used two model selection approaches (AIC_c_ and BIC; Anderson & Burnham, 2002) to compare the fits of the three models for each site × species combination.

**FIGURE A3:**
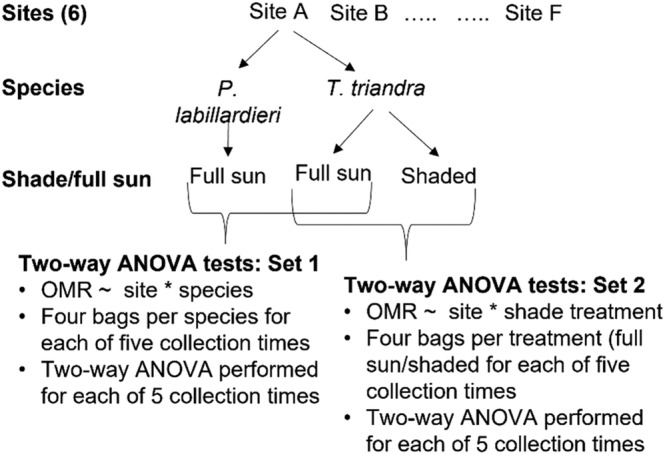
Schematic diagram showing how experimental design relates to two sets of two‐way ANOVAs.

Two sets of two‐way ANOVAs were performed (see Figure A3 for a schematic diagram of our analytical approach). Set 1 tested the individual and interactive effects of sites and species on OMR. Set 2 tested the individual and interactive effects of sites and the shade treatment on OMR. For each set, a two‐way ANOVA was performed for each of the five collection points. For instances where missing data points lead to an unbalanced design, we calculated type II sum‐of‐squares (Langsrud, 2003). We used Kruskal‐Wallis to test the difference in mean Weibull *α* values between shaded and unshaded plots. We used Pearson's correlations to test the relationship between the Weibull *α* constant and mean annual rainfall. We calculated the effect size of the shade treatment as follows:

$$\text{Effect size}=\left(\frac{\bar{m_{u}}-\bar{m_{s}}}{\bar{m_{u}}}\right)\times 100$$

where $\bar{m_{u}}$ and $\bar{m_{s}}$ are the mean mass loss in the unshaded and shaded treatments, respectively. This was calculated for both the fourth and fifth (final) monitoring points. We feel this provides an intuitive effect size, as it expresses the difference in decomposition between unshaded and shaded plots as a percentage.

We calculated the decomposition rate as a percentage of the original mass lost per day by each species at each site during each collection period:

$$\text{Decomposition rate}=\frac{\left(\bar{{\mathrm{OMR}}_{t1}}-\bar{{\mathrm{OMR}}_{t2}}\right)\times 100}{d}$$

where ${\mathrm{OMR}}_{t1}$ and ${\mathrm{OMR}}_{t2}$ are the mean OMR of litter at the beginning and end of a collection period, respectively, and d is the number of days in the collection period. We used Pearson's correlation coefficient to test the relationship between daily decomposition rate and rainfall, solar exposure and mean daily maximum temperature experienced by each site during each collection period. Daily climate data for each site were retrieved from SILO Australian Climate Database (Jeffrey et al., 2001). Cumulative rainfall was determined for each site and each collection period by summing the daily rainfall totals between the time of bag deployment and the collection date. All data analyses were performed in R (R Core Team, 2019) using the packages dplyr (Wickham et al., 2020) and ggplot2 (Wickham, 2016). See Supplementary Data 1 for further explanation on fitting the Weibull models, including R code.

## 3 RESULTS

### 3.1 Model selection results

**FIGURE 2:**
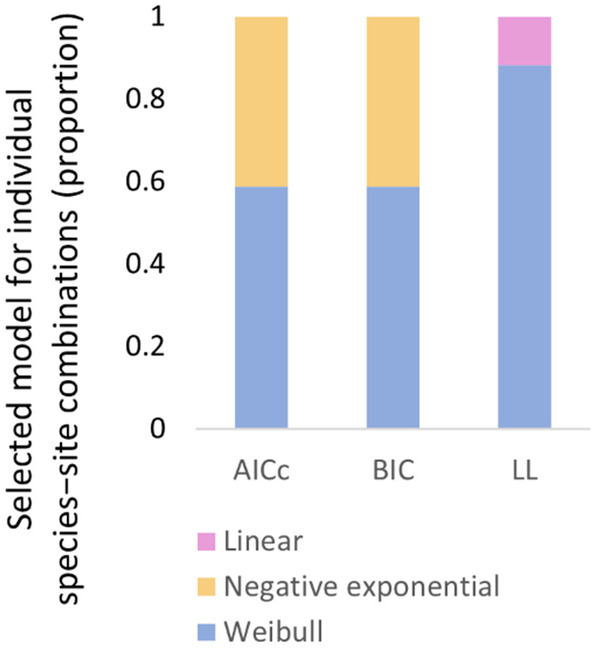
Model selection for 17 site × species and site × treatment combinations, comparing three models (Weibull model, Negative exponential model and a linear model) according to three model selection approaches: the second‐order Akaike Information Criteria (AIC_c_), the Bayesian Information Criteria (BIC) and the log‐likelihood (LL).

The best model, as selected by AIC_c_, varied among the site × species and site × treatment combinations (Figure 2; see Table A1 for full results of the model selection procedure). The Weibull model was selected as the best model for 10 of the 17 combinations, and the negative exponential was selected as the best of 7 of 17 combinations. The BIC selected the same model as AIC_c_ in all but two cases. However, it should be noted that AIC_c_ and BIC each apply a penalty for model complexity to the Weibull model. The log‐likelihood, which provides a measure of the goodness of fit for each model, was highest in Weibull model for 15 of 17 combinations, highest in the linear model for 2 combinations, and in no cases was the log‐likelihood of the negative exponential model higher than the Weibull model. For the remainder of the results, we observe how the Weibull decomposition curve describes the patterns of decomposition, as this was the single best model for summarising decomposition patterns.

### 3.2 Did decomposition vary with site and species?

**FIGURE 3:**
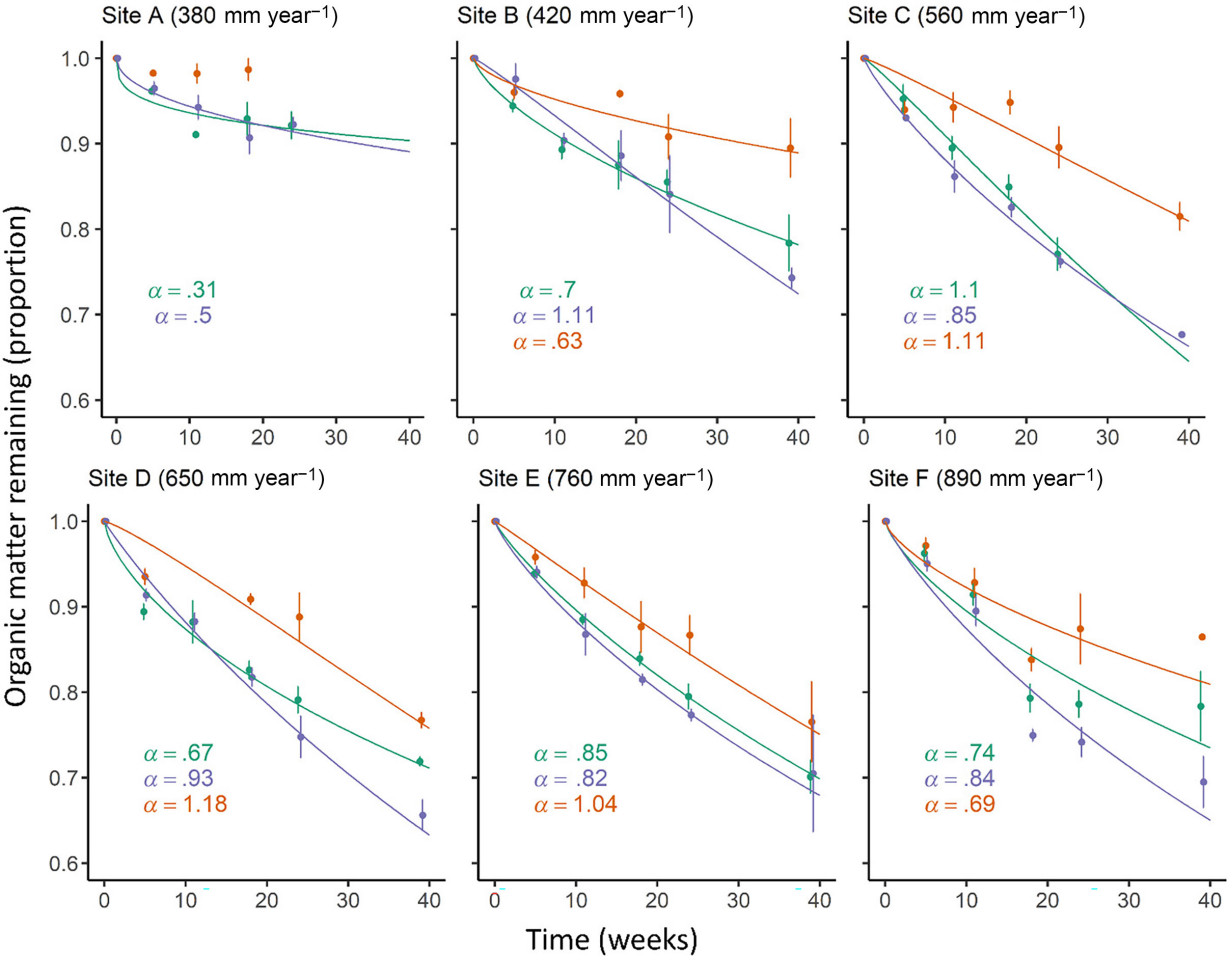
Litter decomposition across a gradient of mean annual precipitation. The trend lines represent Weibull curves fitted to the data. The plotted data points show the mean organic matter remaining (%) for each treatment at each of five collection points over 39 weeks (± standard errors). The three treatments are displayed in green (*Poa labillardierei* var. *labillardierei*), purple (*Themeda triandra* + sun) and orange (*T. triandra* + shade). A Weibull curve could not be fitted to the ‘*T. triandra* + shade’ treatment for Site A; all litterbags for this treatment were destroyed prior to the final sampling period, leading to too few sampling points and a poor fit to the Weibull model. The *α* constant for each Weibull curve is displayed.

The shape of the Weibull decomposition curves varied among sites (Figure 3). For all sites except the driest (Site A), the *α* constant of the Weibull residence models varied between .63 and 1.18, indicating they showed either weak exponential decay or were closer to linear decay (Figure 3). At the driest site (380 mm year^−1^; Figure 3a), *α* was lowest (.31), indicating rapid initial decay and subsequent slow decay; at that site, a high proportion of the total decomposition occurred in the first 10 weeks. However, there was no significant relationship between *⍺* and mean annual rainfall (*R* ^2^ = .06, *p* = .37) or cumulative rainfall during the study period (*R* ^2^ = .13, *p* = .16). The two species had similar decomposition curves at each site, with no significant difference (*p* = .70) in mean *α* values when all sites were pooled (.72 ± .11 for *P. labillardierei*; .84 ± .20 for *T. triandra*).

Total decomposition did not vary significantly between litter types at any of the first four sampling periods (up to 24 weeks) but, by week 39, the total decomposition of *T. triandra* was significantly higher than that for *P. labillardierei* var. *labillardierei* (Table A2; *p* = .027). Conversely, there was a significant difference in total decomposition among the sites at the first four sampling periods (Table A2), but no such difference among the sites at the final monitoring period. There were no site × species interactions at any sampling times (Table A2).

### 3.3 Did climatic conditions at sites affect total decomposition?

**FIGURE 4:**
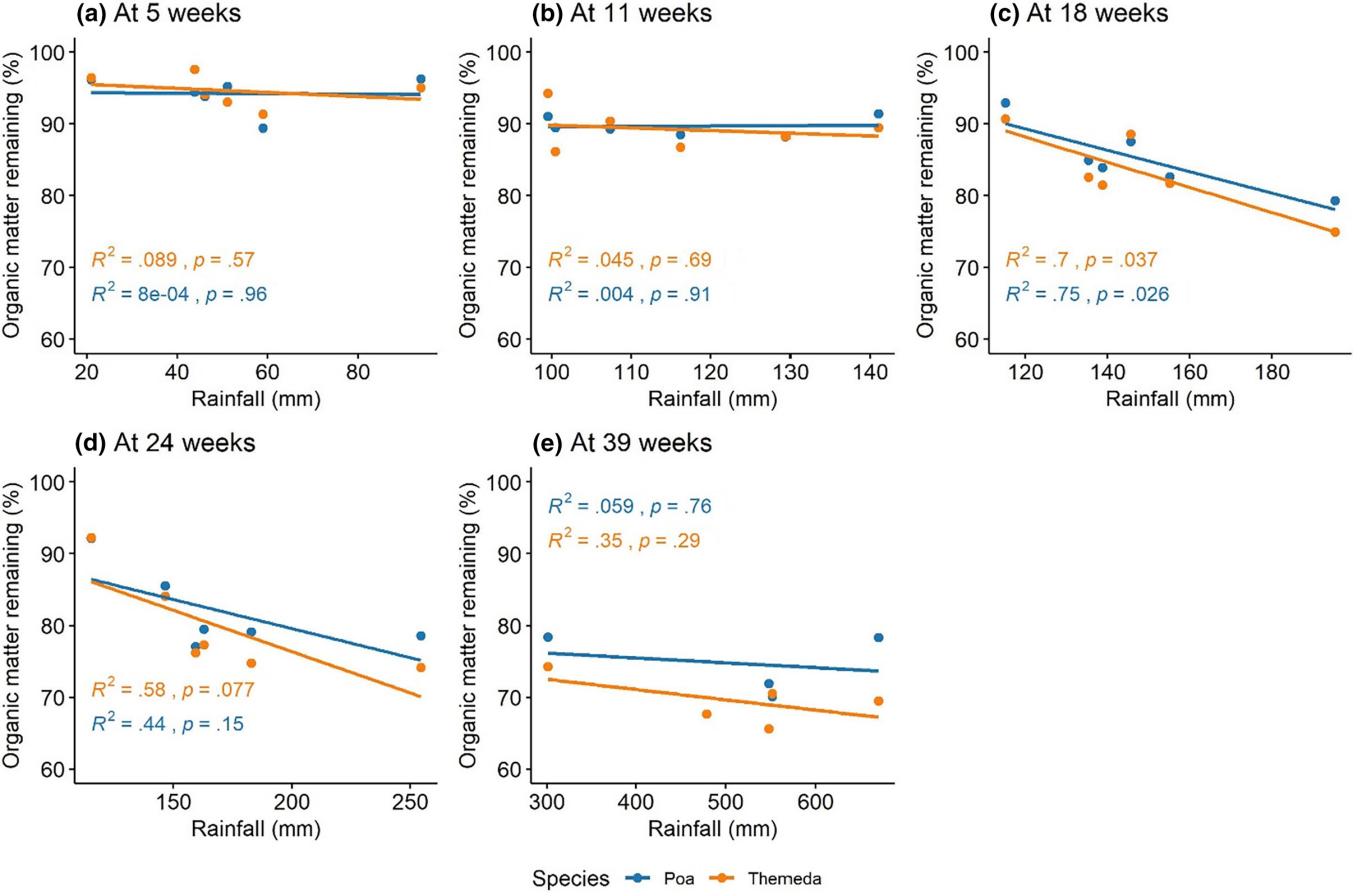
Organic matter remaining (%) versus the cumulative rainfall (mm) for each site after deployment of litter bags. Values for organic matter remaining are means for the treatments at each site.

At the final collection (39 weeks), there was no significant relationship between total rainfall during the study period and OMR (Figure 4e; *P. labillardierei* var. *labillardierei*: *p* = .76; *T. triandra*: *p* = .29). It was only at the third collection—after 18 weeks—that OMR was significantly correlated with ‘rainfall since deployment’ for both species (Figure 4c; *p* = .026 for *P. labillardierei*; *p* = .037 for *T. triandra*).

**FIGURE 5:**
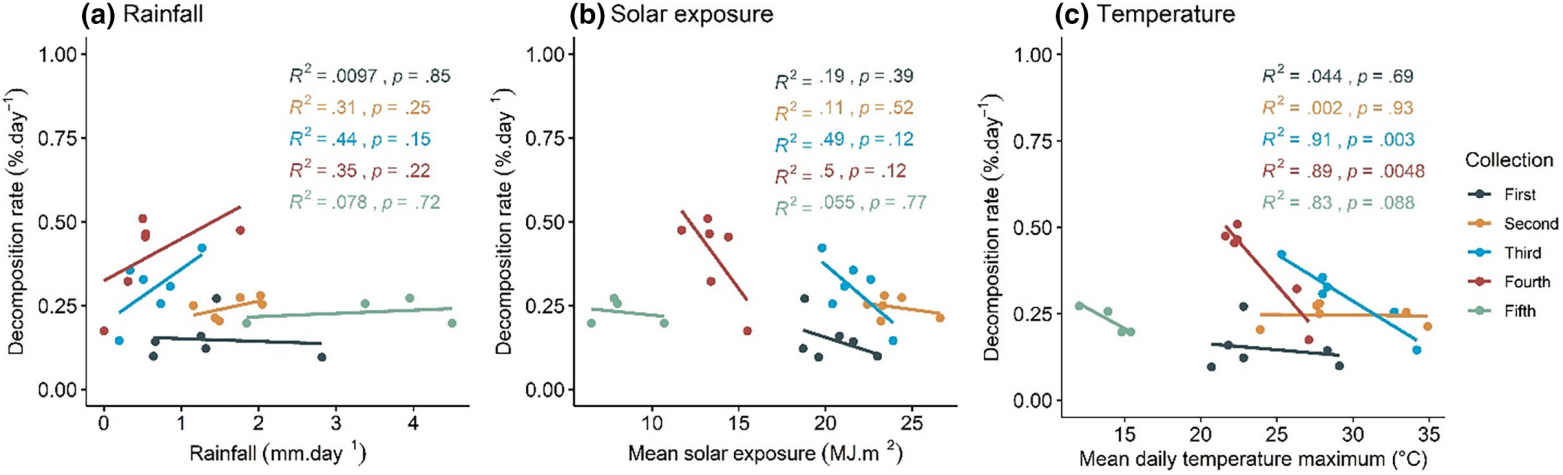
Decomposition rates (percentage mass lost per day) of *Poa labillardierei* var. *labillardierei* during five different time periods and their correlation to (a) rainfall (mm day^−1^), (b) mean solar exposure (MJ m^2^) and (c) mean daily maximum temperature (°C).

The decomposition rate, expressed as a percentage of the original mass lost per day during each collection period, showed no significant relationship to site rainfall (mm day^−1^) or mean solar exposure (MJ m^−2^) at any of the five collection periods (Figure 5). Decomposition was negatively correlated with temperature in the third and fourth collection periods (Figure 5c; *p* = .003 and *p* = .0048 for 18 and 24 weeks, respectively) when the hottest sites showed the least decomposition.

### 3.4 Does the effect of shading vary along the climatic gradient?

**FIGURE A4:**
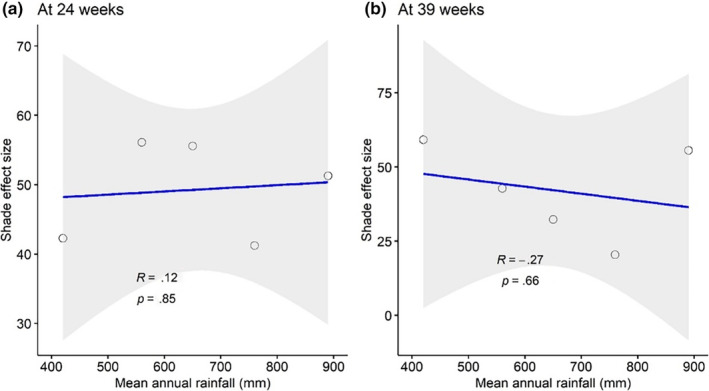
Correlations between mean annual rainfall and the effect size of the shade treatment for the fourth and fifth collection periods (24 and 39 weeks, respectively).

*Themeda triandra* litter decomposition over the study period was significantly lower in shaded plots compared to unshaded plots at each of the five monitoring periods (Table A3) and there were no significant site × shade treatment interactions. At the fourth and fifth monitoring points, shaded plots showed a mean of 50.1% and 38.1% less decomposition, respectively, than unshaded *T. triandra* plots. This reduction was unrelated to the productivity gradient; there was no significant correlation between the effect size of the shade treatment and mean annual rainfall or rainfall during the study period (Figure A4). Regarding the shape of decomposition curves, there was no difference in the mean *α* values (from the Weibull curves) of the shade treatment (.92 ± .11) and those of the other two treatments (*p* = .81). Nevertheless, the four highest *α* values observed (range 1.10–1.18) were from the shade treatment.

### 3.5 How did litter chemistry vary between the two grass species?

Whilst the two species had similar C content (Table 1), *P. labillardierei* var. *labillardierei* had lower N content, and a notably higher C:N ratio than *T. triandra*. *Poa labillardierei* var. *labillardierei* also had lower lignin content and a higher lignin:N ratio than *T. triandra* (Table 1).

**TABLE 1:** Litter chemistry variables for the leaf litter of the two grass species, prior to being decomposed in the field.

Species	Carbon (%)	Nitrogen (%)	Lignin (%)	C:N	Lignin:N
*Poa labillardeiri* var*. labillardierei*	44	0.41	10.6	107.3	25.9
*Themeda triandra*	45	0.79	17.3	57	21.9

*Note*: *n* = 1 for each variable.

## 4 DISCUSSION

Australian grassland litter decomposition is poorly understood relative to grasslands globally, hampering efforts to understand global and local C dynamics. We examined the effects of climate, solar radiation and species differences on litter decomposition. We found the Weibull model more suitable for describing and comparing decomposition trajectories in these systems than the negative exponential model. We found that the effect size of shading on decomposition was not related to the climate gradient and that rainfall was not a strong predictor of litter decomposition. We also observed relatively linear decomposition at most sites, rather than strong negative exponential decomposition characterised by rapid initial decomposition and a slower subsequent decomposition rate. There was some evidence that the grass species *T. triandra* (that had lower C:N and Lignin:N ratios) decomposed faster than *P. labillardierei* var. *labillardierei*. We will discuss how these findings impact our understanding of grass decomposition and the modelling of global decomposition rates.

The grass used in our study was likely to be particularly recalcitrant; when compared to 20 grass species from similar decomposition studies in other continents (Brandt et al., 2010; Henry et al., 2008; Smith & Bradford, 2003; Vivanco & Austin, 2006), the litter in our study contained, on average, half the N content, 1.9 times more lignin content and 3.5 times the Lignin:N ratio (see Table A3). This low N–high‐lignin status may help explain why we observed a relatively linear decomposition trajectory rather than a strong negative exponential relationship (Coleman et al., 2004; Couteaux et al., 1995). Firstly, this may have prevented the rapid initial decay characteristic of negative exponential decay due to a lack of N to fuel decomposition of labile materials. Secondly, this may have aided later‐stage decomposition due to the absence of the inhibitory effect of N on lignin breakdown (Couteaux et al., 1995), which would also work against a trajectory of negative exponential decay. These patterns require further confirmation, but our results suggest that Australian grasslands might experience distinctly different decomposition trends to those reported in the global literature, which are predominantly species and habitats from Europe and the Americas.

The role of photodegradation on decomposition may also help explain the results we observed. Contrary to our expectation, photodegradation did not have a proportionally larger impact on decomposition at the drier sites. Almagro et al. (2016) and Brandt et al. (2007) found photodegradation to be a greater driver of decomposition at sites with decreased moisture availability, though they tested this at drier sites than those tested here. Austin et al. (2016) demonstrated that high‐lignin grasses (like those from our study) are not readily digestible by microbes without a photopriming period in which photodegradation breaks down lignin and allows microbes better access to plant litter carbohydrates. Indeed, in our study, we found that the four highest ⍺ values observed in our Weibull residence models were attributable to the shade treatment, which may indicate a possible lag phase in microbial decomposition due to reduced solar radiation exposure. Despite this, Brandt et al. (2010) found that the decomposition of the grass species *Andropogon gerardii* (Big bluestem), which has a similar Lignin:N ratio to *T. triandra*, was not influenced by UV attenuation at a mesic grassland site (726 mm MAP) but had ~47% greater decomposition without UV attenuation at semi‐arid sites. This contrasts with our observation of a strong and consistent solar radiation effect across the breadth of a similar rainfall gradient. This contrast must be tempered due to low replication and several potential confounding factors. Nevertheless, this discrepancy may indicate a greater role of photodegradation in Australia's high‐lignin grasses, and it suggests we need a better understanding of the interactions among litter quality, climate and decomposition. The results provide some support for the idea that climatic gradients will only be observed in litter that is available for microbial decomposition—that is for carbohydrates that are inaccessible in lignin‐bound cells, higher rainfall will not lead to greater decomposition.

Temperature was not a strong driver of decomposition in our study; this may reflect the relatively narrow range of mean annual temperature differences across our study sites. Previous studies have demonstrated that temperature promotes decomposition. Zhang et al. (2008) report a strong decomposition‐temperature dependency, but this was driven by low decomposition at temperatures below 5°C, whilst the increase in thermal and photodegradation reported by Lee et al. (2012) is driven by temperatures >35°C. It is difficult to disentangle the effect of temperature from other climate variables in our study. Nonetheless, temperature appears to exert an influence upon decomposition that is separate from rainfall effects, and this may warrant further investigation in Australian systems, especially those in cooler (e.g. alpine) and much hotter climates (e.g. tropical savanna).

Site and rainfall emerged as factors that can influence decomposition, although this effect varied temporally. Moisture availability is a prerequisite for microbial activity, and more xeric environments are likely to have lower rates of microbial decomposition (Smith et al., 2010). However, the only significant relationship between cumulative rainfall and litter decomposition we found was transient—in early summer, 11 weeks after deployment—with no such relationship observed at other timepoints. It is possible that these high‐lignin content grasses required a photopriming period that negated temperature–decomposition relationships. Our study highlights the complexity of interactions among abiotic and biotic factors that influence decomposition, which may include the sequence and timing of rainfall and solar exposure.

Our results prompt us to consider the role of grass decomposition in grassland C storage in Australia. Abiotic decomposition represents more rapid C release than biotic decomposition (Austin & Vivanco, 2006). C storage via decomposition is not well‐studied in Australian grasslands, but our study highlights the importance of complex factors, including climate, solar exposure and litter quality on litter decomposition and consequently on the C that they store. It may be that open grass swards behave differently to closed grass swards where shade might prevent photopriming.

## 5 CONCLUSIONS

We found that solar radiation exposure played a considerable role in grass decomposition across a large rainfall gradient and that the patterns of grass decomposition in Australian temperate grasslands are relatively linear. The influence of temperature and rainfall on decomposition was less clear. Further research and understanding of decomposition in these systems is needed, as projected changes in long‐term rainfall and temperature patterns may have important implications for C cycling in grasslands that are currently difficult to predict.

## AUTHOR CONTRIBUTIONS

**Freja E. B. Butler:** Conceptualization (equal); data curation (lead); formal analysis (equal); investigation (equal); methodology (equal); project administration (lead); resources (equal); writing – original draft (equal). **Megan K. Good:** Conceptualization (equal); formal analysis (equal); investigation (equal); methodology (equal); project administration (supporting); resources (equal); supervision (lead); writing – original draft (equal). **John W. Morgan:** Conceptualization (equal); investigation (equal); methodology (equal); supervision (supporting); writing – original draft (equal). **Nick L. Schultz:** Conceptualization (equal); data curation (supporting); formal analysis (lead); funding acquisition (lead); investigation (equal); methodology (equal); resources (equal); supervision (lead); writing – original draft (equal).

### OPEN RESEARCH BADGES

This article has earned Open Data, Open Materials and Preregistered Research Design badges. Data, materials and the preregistered design and analysis plan are available at [https://doi.org/10.25955/c.6490006.v2].

## Data Availability

The data that support the findings of this study is openly available on Figshare at https://doi.org/10.25955/c.6490006.v2.

## APPENDIX 1

**TABLE A1:** Results of model selection.

Site	MAR	Species/treatment	ΔLog‐liklihood			BIC		
			Linear	Neg exp	Weibull	Linear	Neg exp	Weibull
A	380	*Poa*	3.18	4.65	0.00	−79.49	−79.59	−85.85
		*Themeda*	1.92	2.71	0.00	−90.53	−92.04	−94.37
B	420	*Poa*	2.10	3.12	0.00	−81.06	−82.14	−85.25
		*Themeda*	0.02	0.02	0.00	−69.97	−73.12	−70.02
		Shade	0.02	0.02	0.00	−62.31	−64.71	−63.01
C	560	*Poa*	0.00	0.37	0.12	−79.91	−82.17	−79.67
		*Themeda*	5.30	3.91	0.00	−82.90	−88.61	−93.49
		Shade	0.00	0.58	0.57	−84.35	−86.33	−83.20
D	650	*Poa*	5.95	8.97	0.00	−90.42	−87.62	−102.33
		*Themeda*	2.30	1.55	0.00	−90.99	−95.68	−95.59
		Shade	0.00	0.93	0.88	−73.13	−74.22	−71.37
E	760	*Poa*	4.02	2.88	0.00	−94.36	−99.68	−102.40
		*Themeda*	0.00	0.02	0.00	−78.96	−82.30	−78.96
		Shade	2.18	1.66	0.00	−57.46	−61.48	−61.81
F	890	*Poa*	3.02	2.52	0.00	−62.52	−66.71	−68.57
		*Themeda*	1.41	1.29	0.00	−59.36	−62.65	−62.18
		Shade	3.77	2.10	0.00	−66.98	−73.54	−74.51

Site	MAR	Species/treatment	AIC_c_			ΔAIC_c_			Akaike weights		
			Linear	Neg exp	Weibull	Linear	Neg exp	Weibull	Linear	Neg exp	Weibull
A	380	*Poa*	−81.22	−81.01	−87.57	6.36	6.56	0	0.04	0.03	0.93
		*Themeda*	−92.47	−93.59	−96.31	3.84	2.72	0	0.10	0.18	0.71
B	420	*Poa*	−83.2	−83.81	−87.39	4.19	3.58	0	0.10	0.13	0.78
		*Themeda*	−72.11	−74.79	−72.16	2.68	0	2.63	0.17	0.65	0.18
		Shade	−63.8	−65.99	−64.5	2.2	0	1.49	0.18	0.55	0.26
C	560	*Poa*	−81.4	−83.46	−81.16	2.06	0	2.3	0.21	0.60	0.19
		*Themeda*	−84.13	−89.75	−94.73	10.6	4.97	0	0.00	0.08	0.92
		Shade	−86.49	−88	−85.34	1.51	0	2.66	0.27	0.58	0.15
D	650	*Poa*	−92.94	−89.51	−104.84	11.91	15.33	0	0.00	0.00	1.00
		*Themeda*	−93.32	−97.46	−97.93	4.6	0.46	0	0.05	0.42	0.53
		Shade	−74.37	−75.35	−72.6	0.99	0	2.75	0.33	0.54	0.14
E	760	*Poa*	−96.08	−101.11	−104.12	8.04	3.01	0	0.01	0.18	0.81
		*Themeda*	−58.94	−62.77	−63.3	4.35	0.53	0	0.06	0.41	0.53
		Shade	−82.1	−84.57	−82.1	2.47	0	2.47	0.18	0.63	0.18
F	890	*Poa*	−64.85	−68.49	−70.9	6.04	2.41	0	0.04	0.22	0.74
		*Themeda*	−69.49	−75.43	−77.02	7.53	1.59	0	0.02	0.31	0.68
		Shade	−61.08	−64.07	−63.9	2.99	0	0.17	0.10	0.47	0.43
Mean									0.11	0.35	0.54

*Note*: The top half of the table shows ΔLog‐liklihood of each model (i.e. highest log‐likelihood minus log‐likelihood of the other two candidate models) and Bayesian Information Criteria (BIC). The bottom half of the table shows the second‐order Akaike Information Criteria (AIC_c_), ΔAIC_c_ (Difference between AIC_c_ the lowest AIC_c_ in the candidate set; any model with an ΔAIC_c_ < 2 is considered well supported in comparison to the best model) and Akaike weights (which represent the relative likelihood of each model being the best model in the candidate set). The model best supported by AIC_c_ in each candidate set is shaded green.

**TABLE A2:** Results from the two‐way ANOVAs for unshaded plots, testing for (1) differences in organic matter remaining (OMR) among the six sites, (2) difference in OMR between the two species (*Poa labillardierei*, *Themeda triandra*) and (3) site: species interactions in OMR.

Week	Site		Species		Site:Species	
	*F* statistic	*p*‐Value	*F* statistic	*p*‐Value	*F* statistic	*p*‐Value
5	10.101	4.77e‐06***	0.426	.518	2.024	.099
11	3.392	.0136*	0.164	.688	1.324	.277
18	17.762	9.57e‐09***	3.434	.0723	0.566	.725
24	23.526	1.98e‐10***	3.150	.0844	0.459	.804
39^a^	1.665	.194	5.641	.0267*	0.740	.540

*Note*: Statistical significance is highlighted by asterisks (*for .05 > *p* > .01; ** for 0.01 > *p* > .001; *** for *p* < .001; *p*‐values without an asterisk are not statistically significant).

^a^ Tests for which type II sum‐of‐squares was used.

**TABLE A3:** Results from the two‐way ANOVAs for *Themeda triandra* plots, testing for (1) differences in organic matter remaining (OMR) among the six sites, (2) difference in OMR between the shaded and unshaded plots and (3) site: shade treatment interactions in OMR.

Week	Site		Shade treatment		Site:Shade treatment	
	*F* statistic	*p*‐Value	*F* statistic	*p*‐Value	*F* statistic	*p*‐Value
5	8.054	3.53e‐05***	4.89	.0335*	1.161	.347
11	5.019	.008**	17.90	3.17e‐04***	0.738	.540
18	18.606	4.21e‐09***	71.42	4.55e‐10***	0.722	.611
24^a^	2.736	.052	20.99	1.20e‐04***	0.573	.685
39^a^	4.726	.023*	11.66	.003**	0.697	.511

*Note*: Statistical significance is highlighted by asterisks (* for .05 > *p* > .01; ** for .01 > *p* > .001; *** for *p* < .001; *p*‐values without an asterisk are not statistically significant).

^a^ Tests for which type II sum‐of‐squares was used.
